# Cost of shingles: population based burden of disease analysis of herpes zoster and postherpetic neuralgia

**DOI:** 10.1186/s12879-017-2185-3

**Published:** 2017-01-13

**Authors:** Kevin J. Friesen, Dan Chateau, Jamie Falk, Silvia Alessi-Severini, Shawn Bugden

**Affiliations:** 1 College of Pharmacy, Rady Faculty of Health Sciences, University of Manitoba, Winnipeg, MB Canada; 2 Department of Community Health Sciences, College of Medicine, Rady Faculty of Health Sciences, University of Manitoba, Winnipeg, MB Canada

**Keywords:** Herpes zoster, Postherpetic neuralgia, Burden, Epidemiology, Economics, Administrative data

## Abstract

**Background:**

Around 30% of the population will experience herpes zoster (HZ), 10% of whom develop postherpetic neuralgia (PHN). Together, these illnesses produce a significant economic burden to the healthcare system.

**Methods:**

Administrative healthcare data collected over the period of April 1^st^ 1997 to March 31^st^ 2014 were analyzed to determine the healthcare system burden of HZ using direct medical costs. Episodes of HZ were identified using international classification of disease (ICD) codes. Trends in age-adjusted (AA) HZ-rates were analyzed by piecewise-regression. Total annual and per-episode costs were determined for drug treatment, medical care, and hospitalizations within each year.

**Results:**

The incidence of HZ increased by 49.5% from 1997/98 to 2013/14. Piecewise-regression of AA-rates revealed a steady AA-rate of 4.7 episodes/1000 person-years (PY) from 1997/98 to a breakpoint in 2008/09, after which rates began to increase reaching 5.7 episodes/1000 PY in 2013/14.

Drug costs rose significantly (*p* <0.03) from $89.77/episode (95% CI: $82.96, $96.59) to $127.34/episode (95% CI: $117.24, $137.44). Medical costs increased (*p* <0.0001) from $57.98/episode (95% CI; $55.26, $60.70) to $78.84/episode (95% CI; $74.08, $83.61). Hospitalization rates declined from 3.10% in 1997/98 to 1.36% in 2011/12, resulting in cost dropping from $397/episode (95% CI; $284, $511) to $195/episode (95% CI; $129, $260).

Total annual costs of HZ and PHN were $1,997,183 in 2011/12, 4.7% lower than the 1997/98 costs of $2,095,633.

**Conclusion:**

A significant increase in annual number of HZ cases was observed, driven largely by demographic factors. A 21% increase in the AA-incidence reveals changes in HZ rates beyond those expected by population shifts.

The large increase in incidence of HZ, with rising per episode medical and prescription costs were offset by dramatic drops in hospitalization rates, the net effect of which has been to hold the total costs relatively constant. However, the decrease in hospitalization rates slowed over the last half of the study, settling at 1.3% in the last 4 study years. The likely future of HZ burden is one of rising costs, primarily driven by the demographic shifts of an increasing and aging population.

## Background

The varicella zoster virus (VZV) causes varicella zoster (VZ) upon initial infection, affecting the skin and its sensory neurons. As VZ resolves, the virus enters a latent state in neuronal ganglia, remaining there for life [1–3]. Reactivation of VZV, typically within singular ganglia, causes herpes zoster (HZ), a dermatological condition similar in symptoms to VZ but causing moderate to severe pain [4, 5]. A potential complication of HZ is postherpetic neuralgia (PHN), a longer lasting pain syndrome caused by inflammation or virus-induced nerve damage [4–6].

Before a VZ vaccine became available in 1999 (Varivax, Merck Frosst Canada & Co), nearly all persons were infected with VZV, typically as a child. By the age of 40, VZV prevalence was 95–97% [7, 8]. Virtually this entire pre-VZ vaccine population is at risk of developing HZ, with an incidence estimated at between 1.2 and 6.3 cases per 1000 person years (PY), with rates increasing with age. The lifetime prevalence of HZ is between 20–30%, rising to 50% by age 80 [2, 7, 9, 10].

The antiviral drugs acyclovir, valacyclovir, and famciclovir are the cornerstone of HZ treatment and reduce the duration and severity of symptoms, including pain [11]. Treatment should begin within 72 h of the onset of symptoms and continue for 7 days [2, 6, 12]. Antiviral drugs have been reported to account for 50–70% of the drug cost for treated cases of HZ [13–16]. Other drugs used to treat HZ pain include analgesics, opioids, glucocorticoids, and topical lidocaine.

Despite being relatively common, there is no a standard definition of PHN, particularly in reference to the duration of pain which differentiates continuing HZ pain from PHN. One of the more common criteria used is the persistence of pain for 90 days or more post-HZ diagnosis. Studies using this definition report that 5–15% of HZ cases convert to PHN [13–15, 17–19]. PHN is a challenging condition to treat. The Canadian Pain Society consensus statement and the European Federation of Neurological Societies (EFNS) treatment guidelines list tricyclic antidepressants (TCAs), gabapentin, and pregabalin as first line; and opioids or topical lidocaine as second line treatment options for PHN [20, 21].

In randomized controlled trials, the HZ vaccine (Zostavax®, Merck), introduced to Canada in September 2009, was shown to reduce the burden of HZ by 61%, decrease the relative risk of HZ by 51%, and that of PHN by almost 67% [16, 22–24].

A number of recent studies have looked at the burden of HZ-PHN in a variety of health care systems, including Belgium [25], France [26], Germany [14, 27], Greece [28], Italy [13, 29], Spain [18], the United Kingdom [15], and Israel [17]. However, little recent Canadian data have been published. To establish the current burden of HZ and PHN in the setting of a universal healthcare system, and to look at long term trends in their treatment costs, a retrospective, population based study was conducted over the 15 year period from 1997/98 to 2011/12 in Manitoba, Canada.

## Methods

Using an observational, cohort-based methodology, the incidence and burden of HZ and PHN were examined from April 1^st^ 1997 to March 31^st^ 2014 using administrative healthcare data from the province of Manitoba, Canada. Data was obtained from the Manitoba Centre for Health Policy (MCHP), which maintains a data repository containing records of virtually every contact between Manitoba residents and the province’s universal healthcare system [30, 31]. All records are de-identified but contain a unique number that allows researchers to link individual patient records across databases.

Databases used included the Drug Program Information Network (DPIN), a community-pharmacy based prescription processing system that enables submission of online insurance claims by pharmacies, the Medical Services database which contains records of fee-for-service medical claims, Hospital Discharge Abstracts containing summary data of each hospital stay; the Manitoba Immunization Monitoring System (MIMS) database which contains records on vaccines administered in the population, and the Manitoba Health Registry from which population counts and basic demographics on individuals can be obtained.

Cases of HZ were identified using International Classification of Diseases (ICD) diagnostic codes. This method has been shown to be highly selective (positive predictive value 93%) and sensitive (97.5%) [32]. Individuals with one or more ICD-9-CM (Clinical Modification) codes starting with ‘053’, or ICD-10-CA (Canadian enhancement) codes starting with ‘B02’ were classified as HZ cases, with episodes starting on the date of the first code. HZ was considered to have converted to PHN when medical services claims or prescriptions for HZ were received 90 days or more after diagnosis.

Multiple episodes of HZ were allowed provided two conditions were met: 1) a minimum of 2 years had elapsed since the start, and 2) a minimum of 180 days had elapsed since the last HZ ICD code of the preceding episode. To avoid misclassification of prevalent episodes, episodes with start dates prior to April 1^st^ 1997 were discarded. Episodes in individuals under 20 years of age were excluded from the analysis to avoid misclassification of potentially miscoded VZ cases. Furthermore, to avoid misclassification of physician visits regarding HZ vaccine as episodes, the MIMS database was searched for HZ vaccination records. As MIMS does not capture all HZ vaccinations we also used DPIN to search for vaccine prescriptions, and medical claims to find vaccination tariff codes, using these as surrogate markers of vaccination. Any HZ episode starting within 30 days of vaccination was excluded.

Pharmacotherapy was assessed using DPIN. Prescriptions for acyclovir, valacyclovir, and famciclovir dispensed in the first 30 days of an episode were classified as HZ antiviral treatment. DPIN was searched by Anatomical Therapeutic Chemical (ATC) class. Prescriptions for nabilone (ATC class A04AD), local anesthetics (D04A), systemic corticosteroids (H02A), NSAIDS (M01), opioids (N02A), ASA, acetaminophen (N02B), anticonvulsants (N03A), and antidepressants (N06A) were categorized as HZ pain prescriptions, provided that several conditions were met: 1) treatment of pain with any drug class began within 90 days following diagnosis; 2) use of a drug class was incident to diagnosis; and 3) use after the later of 90 days post-diagnosis and date of last ICD code for HZ was continuous. Use within a drug class was considered incident if less than 30 of the 90 days pre-diagnosis had prescription coverage from that class. Continuous use was defined as the ongoing dispensing of prescriptions of the above-named ATC classes, with no gaps between consecutive prescriptions greater than 200% of the duration of the earlier prescription.

All costs were adjusted for inflation to 2013 Canadian dollars using the Statistics Canada consumer price index.

HZ episodes were further stratified into two subcategories based on PHN status: HZ episodes with no diagnosis of PHN (HZ-only), and HZ episodes that converted to PHN (HZ-PHN). Costs were analyzed across all HZ episodes, and then within each stratum separately. The Manitoba Pharmacare fiscal year (April 1^st^ to March 31^st^) was used for analysis over time. All costs and events were considered to have been incurred in the fiscal year of diagnosis.

Due to observation time required, two separate reporting periods are used. Epidemiology is reported from 1997/98-2013/14, as only a single observation is required to diagnose HZ, and no follow-up time is required. For burden analysis, two years of observable time is required to capture costs accruing over episodes, especially for HZ-PHN. Therefore, to capture all costs associated with each episode and allow for equal follow-up time we only report burden results of episodes initially diagnosed before the end of the 2011/12 fiscal year.

### Statistical analysis

The number of episodes and incidence rate of HZ, and PHN conversion rates were calculated for each study year. We calculated annual incidence rates (per thousand person years) using population counts from the Manitoba Health registry for that particular year. The annual age-adjusted incidence rate was then determined by calculating age group-specific rates, and using them to directly standardize the overall incidence rate, with the 1997 Manitoba age-distribution as our reference structure. A segmented regression analysis was conducted to examine an apparent change in incidence of HZ. Ordinary least squares (OLS) regression analysis was used for trend analysis of costs and utilization. Due to non-linearity and high variance a *t*-test was used to compare annual hospital costs over the first (1997/98-2003/04) and last (2004/05-2011/12) halves of the study period.

SAS® version 9.4 (SAS Institute, Cary, NC) and Microsoft Excel 2013® (Microsoft Corporation, Redmond WA) were used for data analysis. Approvals were granted by the University of Manitoba Health Research Ethics Board and the Manitoba Health Information Privacy Committee.

## Results

### Epidemiological analysis: 1997/98 to 2013/14

A total of 73,886 episodes of HZ were diagnosed between 1997/98 and 2013/14, an overall crude incidence rate of 4.99 cases/1000 person-years (PY). As we have previously reported [33], a sustained upward trend in the annual numbers of HZ episodes was observed across the study period (Table 1). There were 5,746 episodes of HZ identified in 2013/14, a 49.5% increase from 1997/98.

**Table 1:** Overall health system burden of herpes zoster and postherpetic neuralgia in Manitoba, Canada

FY Year	Cases		Medical Services		Hospitalization		Drugs		Annual Totals	
	HZ	PHN	Visits	Cost	Stays	Cost	Rxs	Cost	Overall	PHN Only
1997/98	3844	331	9738	$222,864	119	$1,527,684	7842	$345,085	$2,095,633	$625,970
1998/99	3781	349	9311	$216,567	94	$947,462	8139	$346,495	$1,510,524	$521,352
1999/00	3941	367	9645	$240,928	97	$1,176,332	9927	$498,362	$1,915,623	$852,724
2000/01	3954	399	9981	$252,837	80	$978,360	9758	$475,111	$1,706,307	$622,177
2001/02	4060	429	10218	$268,900	95	$1,171,142	10548	$532,201	$1,972,243	$809,190
2002/03	4126	410	10549	$262,471	78	$1,116,598	10350	$523,136	$1,902,205	$507,749
2003/04	4070	375	10191	$275,353	83	$1,097,938	10169	$511,907	$1,885,197	$820,833
2004/05	4087	379	10291	$277,410	61	$621,227	9578	$504,787	$1,403,424	$406,629
2005/06	4131	432	10828	$286,785	49	$443,617	10507	$543,196	$1,273,598	$455,895
2006/07	4143	377	10849	$297,755	67	$924,959	10551	$530,623	$1,753,338	$473,495
2007/08	4207	377	11360	$314,783	72	$704,245	10679	$512,113	$1,531,142	$469,445
2008/09	4295	440	11615	$310,292	59	$809,855	12265	$555,622	$1,675,768	$400,491
2009/10	4584	427	12561	$355,407	58	$582,423	12456	$553,298	$1,491,129	$559,819
2010/11	4622	449	12998	$363,637	58	$627,314	12217	$578,973	$1,569,924	$633,618
2011/12	4984	497	13945	$392,947	68	$969,563	14439	$634,674	$1,997,183	$768,050

*Abbreviations*: FY fiscal year, HZ herpes zoster, PHN postherpetic neuralgia, Rxs prescription dispensations

Results were summarized by fiscal year with individuals episodes data considered to have occurred in year of diagnosis

All costs have been adjusted to 2013 Canadian dollars using Statistics Canada consumer price index

**Fig. 1:**
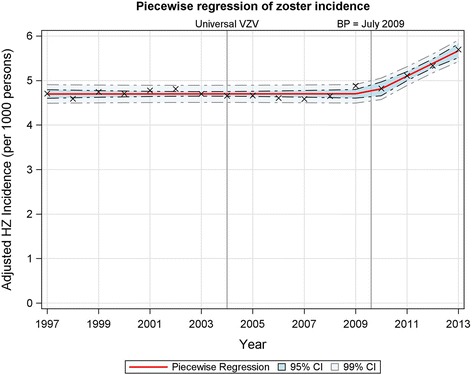
Piecewise regression on age adjusted incidence of herpes zoster. The age adjusted incidence of herpes zoster was calculated using 1997 as the reference year for age standardization. A highly significant breakpoint was found in July 2009 (*p* < 0.0001). Varicella zoster vaccinations were added to the routine childhood vaccination schedule in 2004. Abbreviations: VZV, varicella zoster vaccinations; BP, breakpoint; HZ, herpes zoster; CI, confidence interval

To adjust for the effects of an increase in the size and age distribution, the age-adjusted (AA) incidence rate was calculated and plotted (Fig. 1), revealing an abrupt change in the observed trends in AA-rates starting around 2009/10. Piecewise regression on the AA-incidence revealed a breakpoint in July 2009 (F_(3,13)_ = 59.6 *p* < 0.0001). Prior to 2009/10, the incidence of HZ remained relatively steady (*p* = 0.96) at 4.70 cases/1000 PY (95% confidence interval (CI): 4.65, 4.75). Starting in 2009/10 the AA-incidence began increasing on average by 0.29 cases/1000 PY/year (95% CI: 0.20, 0.37), reaching 5.70 cases/1000 PY in 2013/14, a 21% increase from pre-2009/10 level.

### Burden of disease analysis: 1997/98 to 2011/12

#### Drug utilization trends

Trends in drug utilization, medical claims cost, hospitalizations, and total cost were examined over the period from 1997/98 to 2011/12.

Antiviral treatment rates increased from 41.7 to 62.9% (F_(1,13)_ = 220 *p* < 0.0001 R^2^ = 0.94) over this period. The cost per treated episode dropped from $139.61 to $96.20 due to the introduction of generic antiviral drugs. The mean cost of pain treatment per episode increased by over 111% for HZ, rising from $31.59 (95% CI: $25.35, $37.84) to $66.81 (95% CI: $56.84, $76.79), and by 94% for HZ-PHN, from $291 (95% CI: $225, $358) to $566 (95% CI: $478, $655) over the same interval. On average, HZ-PHN episodes were responsible for 83% of pain-related drug costs, a proportion that remained unchanged over time (*p* = 0.57).

**Fig. 2:**
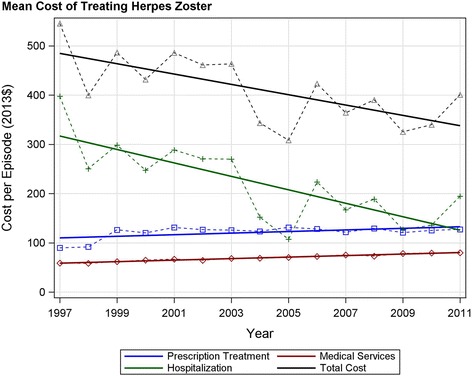
Cost per episode of herpes zoster by treatment modality. The mean cost of treating herpes zoster was determined within each year and regression analysis performed (*solid lines*). Significant trends were found for all modalities: prescription and medical costs *p* < 0.0001; hospitalization *p* < 0.0002. All costs have been adjusted to 2013 Canadian dollars using Statistics Canada consumer price index

The mean cost for all drug treatment rose significantly (F_(1,13)_ = 6 *p* < 0.03 R^2^ = 0.32), from $89.77 (95% CI: $82.96, $96.59) to $127.34 (95%CI: $117.24, $137.44). This increase occurring within the first three study years (Fig. 2). There was a significant upward linear trend in the total annual drug cost (F_(1,13)_ = 30.4 *p* < 0.0001 R^2^ = 0.70), a result of the increase in the number of HZ cases and the rise in mean per episode cost.

#### Trends in medical care

An upward trend in utilization of medical services was observed (F_(1,13)_ = 59.5 *p* < 0.0001 R^2^ = 0.82), The number of medical claims per episodes of zoster rose slightly from 2.53 claims/episode (95% CI: 2.41, 2.65) to 2.80 claims/episode (95% CI: 2.67, 2.93), while the costs per medical claim rose 23% (F_(1,13_ = 59.9, *p* < 0.0001 R^2^ = 0.82). Together, these trends produced a linear increase in the per episode cost (F_(1,13_ = 48.0 *p* < 0.00001 R^2^ = 0.76) from $57.98/episode (95% CI: $55.26, $60.70) in 1997/98, to $78.84/episode (95% CI: $74.08, $83.61) in 2011/12 (Fig. 2). The effect of these trends is amplified by the increase in HZ cases resulting in a significant increase in total annual medical costs (F_(1,13_ = 182.0 *p* < 0.0001 R^2^ = 0.93) (Table 1).

#### Hospitalization trends

The cost per HZ related hospitalization did not change significantly over the study period with a mean of $12,038/ hospitalization (95% CI: $11,068, $13,007). The overall mean length of stay was 13.95 days (95% CI: 12.71, 15.19), this also did not change significantly (1997/98 vs. 2011/12 t = −1.62, *p* = 0.11). There was a sharp drop in the rate of HZ-related hospitalization across the study period (F_(1,13)_ = 49.6 *p* < 0.0001 R^2^ = 0.79), from 3.10% in 1997/98 (119 hospitalizations) to 1.36% in 2011/12 (68 hospitalizations). This resulted in the mean per episode cost of hospitalization dropping significantly (F = 25.0 *p* = 0.0002 R^2^ = 0.62) from $397 (95% CI: $284, $511) to $195 (95% CI: $129, $260), a decline of 51.0%.

Hospitalization costs accounted for 48.5% of total costs in 2011, down from 72.9% in 1997/98. The mean annual cost over the first half of the study (1997/98-2003/04) was $1,145,074 (95% CI: $968,817, $1,321,331), significantly higher than the mean cost of $710,400 (95% CI: $560,336, $860465) seen over last half of the study (2004/05-2011/12) (t_(13)_ = 4.55, *p* = 0.0005).

#### Trends in total cost

**Fig. 3:**
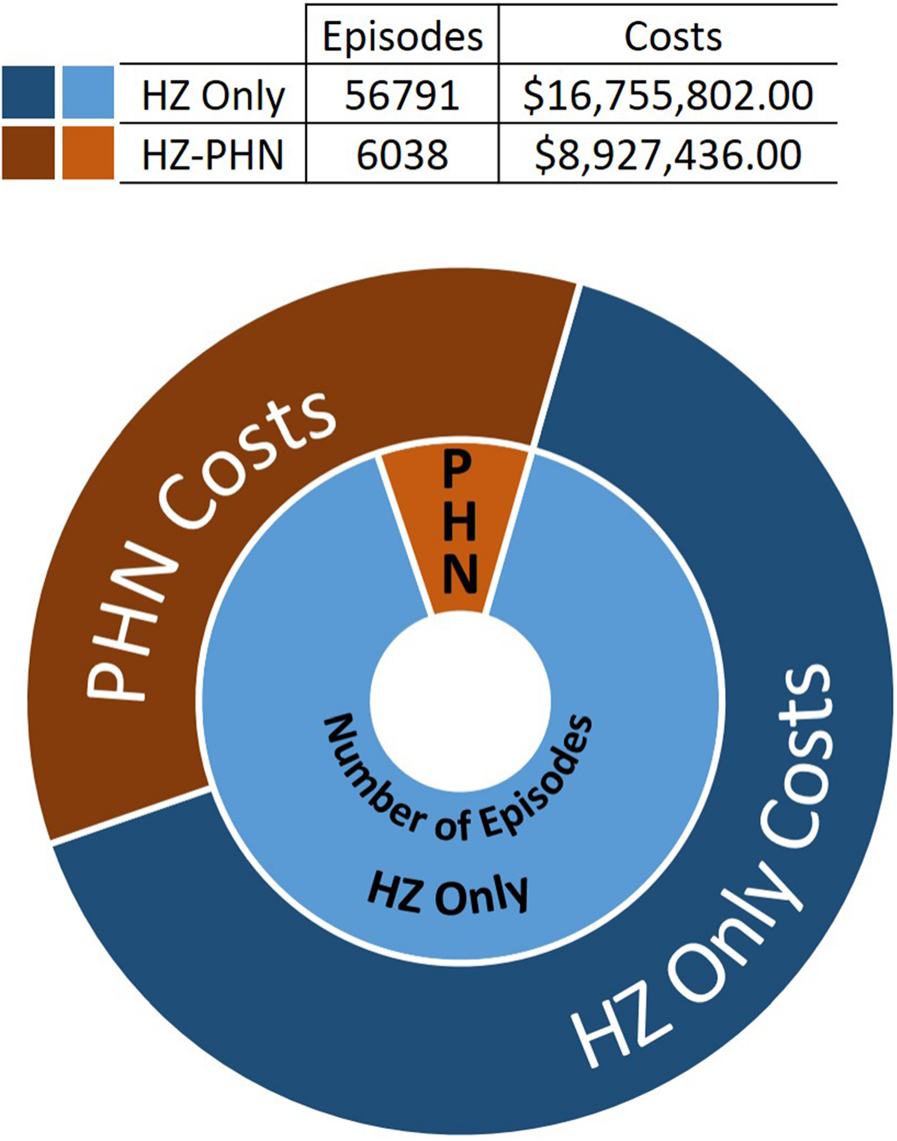
Overall Burden of Herpes Zoster: 1997/98 to 2011/12. The total economic and epidemiologic burden of herpes zoster was determined for the period from 1997/98 to 2011/12 and are shown in the above figure. The inner circle shows the number of episodes broken down by post-herpetic neuralgia (PHN) status, with the orange portion representing episodes in which it occurred, while the outer ring breaks down the total costs in 2013 Canadian dollars. While PHN accounted for less than 10% of all episodes, it was responsible for 35% of total costs

The annual total health care costs of treating HZ are shown in Table 1. Linear regression analysis revealed no significant trend across the study period (*p* = 0.25) with the total health system burden of HZ and PHN being 4.7% lower in 2011/12 than in 1997/98. While less than 10% of HZ episodes converted to PHN, they accounted for almost 35% of total costs overall (Fig. 3).

### Burden of herpes zoster in 2011/12

There were 4984 diagnosed episodes of HZ in 2011/12, an incidence of 5.3 episodes/1000 PY. Of these, 497 went on to develop PHN, a conversion rate of 10.0%. Hospitalization accounted for 49% of total cost, medical services for 20%, and prescription drug costs for 32%. Although PHN occurred in only a tenth of all episodes, they were responsible for 41.6% of hospital costs, 21.3% of medical cost, and 49.7% of drug costs. Overall, HZ-PHN episodes accounted for 38.5% of total HZ-related costs.

The mean cost of an episode of HZ in 2011/12 was $401 (95% CI: $318, $484). The mean cost for HZ that converted to PHN (HZ-PHN), including the cost of treating PHN, was $1614 (95% CI: $1009, $2220). HZ episodes not associated with PHN (HZ-only) had a mean cost of $266 (95% CI: $204, $329), 16.5% of HZ-PHN episodes.

The mean cost of treating an episode of HZ with prescription drugs in 2011/12 was $127.34. Of this, 52.5% was from the treatment of pain with a mean cost of $66.81 (95% CI: $56.84, $76.78), the remainder arising from antiviral treatment at a mean of $60.53 (95% CI: $60.43, 59.92). For HZ-PHN episodes the mean drug cost was $635 (95% CI: $547, $723) with pain treatment accounting for 89% of this cost at $566/episode (95% CI: $478, $655). HZ-only episodes had a mean drug cost of $71.12 (95% CI: $69.18, $73.05), over 83% of which was due to antiviral prescriptions treatment.

The average HZ episode in 2011/12 resulted in 2.80 (95% CI: 2.67, 2.93) medical claims for a mean cost of $78.84 (95% CI: $74.08, $83.61). The average HZ-PHN episodes resulted in 5.57 claims (95% CI: 4.86, 6.29) with a mean cost of $168.30 (95% CI: $128.79, $207.81), while the average HZ- only episodes resulted in 2.49 claims (95% CI: 2.38, 2.60) with a mean cost of $68.93 (95% CI: $66.08, $71.79).

HZ-related hospitalization was uncommon, and occurred in 1.36% of episodes in 2011/12 with a mean cost per stay of $14,258/hospitalization (95% CI: $9,461, $19,056). HZ-PHN episodes were more frequently hospitalized at a rate of 5.0%, whereas HZ-only episodes had a hospitalization rate of 1.0%. There was no significant difference in the cost per hospitalization between HZ-PHN and HZ-only episodes so these results were combined. The cost of hospitalization averaged across all HZ episodes was $194.54 (95% CI: $115.90, $273.17).

## Discussion

This study explored the burden of HZ in terms of healthcare system costs. A significant increase in the incidence of HZ, independent of demographic shifts in the population, was found to begin in 2009/10. The medical cost per episode increased, as did total annual costs. There was an increase in the per episode cost of drug treatment over the first 3 years of study period, after which costs stabilized and remained relatively constant. The combination of these trends in per episode costs arising from medical care and drug treatment were multiplied by the increase in the annual incidence of HZ, causing total outpatient costs to increase. However, this increase was offset by the dramatic drop in rates of hospitalization and the resulting decrease in hospital costs.

Although hospitalization is uncommon in HZ patients, at a cost of $12,000 per stay it has a disproportionate impact on the total burden. In 1997/98, hospital costs accounted for almost three quarters of total HZ costs, this proportion had dropped to 40–50% over the final study years. This is a result of a dramatic change in rates of hospitalization, which dropped from 3.10% in the first study year to 1.36% in the last year. However, it appears that this is the floor to hospitalization rates, as the downward trend appears to have ended midway through the study.

The same is not true regarding the trends seen in number of cases, prescription drug costs, and medical service utilization. The number of HZ cases increased by 50% from 1997/98 to 2013/14, largely driven by demographic changes. The cost per episode of medical care has also seen sustained increases. Increasing costs of pain treatment were offset by drops in antiviral costs leaving the per-episode drug cost steady, however, the increasing number of episodes has resulted in increasing total drug costs. These trends do not appear to be slowing down, and if hospitalization rates have indeed plateaued, it may be the case that the burden of zoster will increase in the future.

The 49.5% increase in the crude incidence of HZ, which began to rise after 2009/10, is one of the most interesting results of this analysis. A significant portion of this increase is related to demographic changes in the province, namely, an increase in the population coupled with an upward shift in the age distribution. However, after controlling for these factors by calculating the incidence rate, and adjustment of rates to a standard age distribution, there remained a significant 21% increase in HZ left to be explained.

One possible explanation is based on exposure to wild VZV, circulating in the population, acting as an exogenous boost to HZ cell-mediated immunity in latently infected adults [34–36]. It has been proposed that an unintended consequence of VZ vaccination programs could be an increase in HZ rates. However, researchers have found mixed evidence when searching for such an effect at the population level [34, 37–42]. A study published using data from Alberta, Canada also found an increase in the rates of HZ. However, they reported that this trend preceded the introduction of the VZ vaccine [39]. Another Canadian study looking at this same issue in Ontario found no increase in HZ rates [40]. However that study ended just as the upward trend reported here began. While this study was not designed to examine such a relationship, it is interesting to note that the VZ vaccinations were added to the routine schedule of universally provided childhood vaccinations in 2004, just 5 years before the increase began. Uptake rates rose quickly as the program was rolled out across various age groups. By 2007/08 over 26% of all children under 18 had been vaccinated, and 76% of those who were scheduled to be vaccinated were [43].

While the HZ vaccine was introduced in 2009, it has not been covered by the provincial insurance drug program and has had limited uptake in Manitoba. As such, it is not possible to assess the potential impact of the HZ vaccine on the economic burden of HZ in Manitoba. However, some jurisdictions are now providing the HZ vaccine free of charge to those over age 65 [44]. In the future the impact of wide spread use of the HZ vaccine in these jurisdictions may allow a full assessment of the impact of vaccination on HZ burden. An important limitation to our results on the burden of HZ is that only direct medical expenses have been considered. There are other sources of burden for both patient and society outside of this narrow focus, perhaps the most important being changes to individuals’ quality of life, especially for those individuals affected by PHN. Societal costs such as lost productivity, disability payments, and opportunity costs are also outside of the scope of this study. However, this analysis has numerous strengths, including the fact that costs are not estimated based on models nor inferred from a sample but rather directly measured across the entire provincial population. This study examined 15 years of healthcare data, allowing us to see changes over an extended period of time.

## Conclusion

This study explored the burden of HZ in terms of healthcare system costs. A significant increase in the incidence of HZ, independent of demographic shifts in the population, was found to begin in 2009/10. The medical cost per episode increased, as did total annual costs. Per-episode drug costs remained level, with increased mean costs of treating pain offset by decreases in antiviral cost per treatment. The combination of increase per-episode medical costs, and constant per-episode drug costs were multiplied by increasing numbers of episodes, causing total outpatient costs to increase. However, this was offset by a dramatic drop in rates of hospitalization.

## Abbreviations

AA: Age-adjusted
ASA: Acetylsalicylic acid
ATC: Anatomical therapeutic chemical classification
CI: Confidence intervals
DPIN: Drug program information network
HZ: Herpes zoster
HZ-only: Herpes zoster without the involvement of postherpetic neuralgia
HZ-PHN: Herpes zoster with postherpetic neuralgia
ICD: Using international classification of disease
ICD-10 -CA: ICD version 10, Canadian enhancement
ICD-9-CM: ICD version 9, clinical modification
MCHP: Manitoba centre for health policy
MIMS: Manitoba immunization monitoring system
NSAIDS: Non-steroidal anti-inflammatory drugs
OLS: Ordinary least squares regression
PHN: Postherpetic neuralgia
PY: Person-years
VZ: Varicella zoster
VZV: Varicella zoster virus
